# Supplementary material for: The wave-particle duality of corporate financial metrics
Source: PLoS One. 2025 Nov 21;20(11):e0336976. doi: 10.1371/journal.pone.0336976 (PMC12637925; doi:10.1371/journal.pone.0336976)
Supplement: Attachment — Submitted filename: Comments to the authors_PONE-D-25-40877.pdf [file pone.0336976.s002.pdf]

Manuscript ID: PONE-D-25-40877

Title: Cracks in the Foundation: The Hidden Toll of Intergroup Conflict on Departmental Performance in Higher Education Islamabad

Comments to the Author

Dear Author/s

Thank you for submitting your manuscript entitled *"The Wave-Particle Duality of Corporate Financial Metrics"* to *PLOS ONE*. The study addresses an important issue in corporate finance and has the potential to make a meaningful contribution to the literature. Both reviewers recognized the significance of your work, though they emphasized the need for improvement.

Before the manuscript can be considered for publication, a few issues must be addressed:

- The introduction does not adequately connect the wave–particle duality concept to corporate financial indicators. The title promises a strong conceptual framing, but the explanation of quantum dynamics (fluctuations, abrupt jumps) is brief. The review needs to be expanded with recent and relevant studies (particularly post-2020) to establish the current gap in the literature. So, additional clarity on theory-based and theoretical contributions is required to establish the novelty of this research.
- The manuscript uses both ROE and ROA for firm performance, but the rationale is not well explained. ROE might be sufficient for performance analysis in some cases, particularly when comparing similar companies in the same industry, examining long-term trends, and understanding its components. Authors should clarify why both are necessary
- Draw a clear and concise hypothesis for testing.
- While panel data is established, the manuscript should describe the population, sampling frame, and chosen sampling method. Reliance on annual panel data may underrepresent high-frequency dynamics and systemic shocks. Reviewer 2's concern about missing volatility patterns is valid and should be discussed as a limitation.
- The multivariate regression (Model 10) shows an adjusted R^2 of 0.430, which reflects relatively modest explanatory power. Authors should acknowledge this limitation and discuss possible model improvements.
- Analysis and discussion mainly reiterate findings. A stronger theoretical foundation is required.

We encourage you to carefully address the points above, along with the feedback from both reviewers, in preparing a revised version.

We look forward to receiving your revised version.

Decision: ***Minor Revision***

Academic Editor
