# Supplementary material for: The wave-particle duality of corporate financial metrics
Source: PLoS One. 2025 Nov 21;20(11):e0336976. doi: 10.1371/journal.pone.0336976 (PMC12637925; doi:10.1371/journal.pone.0336976)
Supplement: Attachment — Submitted filename: Response to Reviewers..docx [file pone.0336976.s005.docx]

**Response to the Academic Editor and Reviewers**

(Consistent with the Revised Manuscript)

Dear, Academic Editor and two Reviews,

We sincerely thank the Academic Editor and both reviewers for their careful evaluation and insightful comments.

All suggestions have been thoroughly addressed in the revised manuscript, with changes highlighted in red.

Below we provide a detailed, point-by-point response.

**A. Response to the Academic Editor**

**Editor Comment 1**

The introduction does not adequately connect the wave–particle duality concept to corporate financial indicators. The title promises a strong conceptual framing, but the explanation of quantum dynamics (fluctuations, abrupt jumps) is brief. Please strengthen theoretical clarity and update references with post-2020 studies.

Response:

We have substantially expanded the theoretical explanation in Section 1 (Introduction) and Section 2 (Literature Review).

The revision explicitly distinguishes ROA as the “wave” component—representing continuous operational efficiency—and ROE as the “particle” component—representing discrete policy- or capital-driven shocks.

In addition, we incorporated recent co-authored studies by Alharbi et al. (2025) on financial-policy uncertainty and green innovation to reinforce the post-2020 relevance of the framework.

We also retained Aït-Sahalia & Jacod (2012) and Aït-Sahalia, Jacod & Li (2012) as foundational methodological references on jump-diffusion modeling, forming the theoretical bridge between financial discontinuities and the proposed dual-state model.

**Editor Comment 2**

The manuscript uses both ROE and ROA for firm performance, but the rationale is unclear. Please clarify why both are required.

Response:

This point is clarified in Section 2 (Literature Review).

ROA reflects continuous operational changes (“wave”), whereas ROE captures discrete leverage and valuation shocks (“particle”).

Employing both indicators allows the model to describe the coexistence of continuous and discontinuous behaviors that characterize real-world firm dynamics.

**Editor Comment 3**

Draw clear and concise hypotheses for testing.

Response:

Two explicit hypotheses have been added at the end of Section 1, and a new Figure 1 (Hypothetical Framework) summarizes how continuous fluctuations (σₜ) and discrete jumps (Iₜvₜ) jointly influence profitability under the wave–particle duality paradigm.

**Editor Comment 4**

Describe the population, sampling frame, and chosen sampling method. Annual data may underrepresent volatility; please discuss this limitation.

Response:

Section 4 (Empirical Analysis) now specifies that the sample includes 805 A-share manufacturing firms (2009–2024) from the CSMAR database, verified with audited annual reports.

The Conclusion explicitly acknowledges that annual data may smooth high-frequency volatility and indicates that future studies will adopt quarterly or event-window datasets to capture short-term dynamics.

**Editor Comment 5**

Model 10 shows adjusted R² = 0.430, which is modest. Please acknowledge this limitation.

Response:

Acknowledged in Section 5 (Results and Discussion) and reiterated in the Conclusion.

The moderate R² reflects the complexity of nonlinear financial processes and the realistic balance between model generality and explanatory power.

**Editor Comment 6**

A stronger theoretical foundation is required to link findings and the wave–particle duality framework.

Response:

The theoretical linkage has been strengthened throughout Sections 2, 5, and 6 (Conclusion).

We clarify that wave–particle duality is used as a conceptual modeling metaphor rather than a physical analogy, unifying continuous (ROA) and discrete (ROE) behaviors within corporate financial systems.

**B. Response to Reviewer #1**

**Comment 1**

Add more recent, directly relevant studies (Alharbi et al., 2025; Aït-Sahalia & Jacod 2012).

Response:

Done in Section 2 (Literature Review).

We added Alharbi et al. (2025) as a recent co-authored contribution on policy uncertainty and innovation dynamics and maintained Aït-Sahalia & Jacod (2012) and Aït-Sahalia, Jacod & Li (2012) as key methodological references on jump processes.

Together these strengthen both the temporal relevance and theoretical continuity of the literature base.

**Comment 2**

Provide a more integrated discussion of empirical results in relation to the policy context.

Response:

Expanded in Section 5 (Results and Discussion).

ROE/ROA jump frequencies are now explicitly linked to major Chinese policy episodes—2015 deleveraging, 2018 environmental reforms, and 2020–2021 pandemic shocks—demonstrating how macro shocks generate “particle” states while stabilization restores “wave” dynamics.

**Comment 3**

Include clear definitions for all variables directly in table notes.

Response:

In Table 4 of the original paper, we provide the definitions of all variables that enter the regression model, but to facilitate readers' reading, we provide additional explanations for each variable that appears in the regression results in Tables 6 and 7.

**Comment 4**

Strengthen the explicit link between the main findings and the wave–particle duality framework.

Response:

Clarified in Sections 5 and 6 (Conclusion), where ROA is interpreted as wave-like and ROE as particle-like behavior.

This explicit mapping empirically validates the dual-state dynamics central to the proposed framework.

**C. Response to Reviewer #2**

**Comment 1**

The predictive models still struggle with extreme outlier years, limiting robustness.

Response:

Acknowledged in Section 5 (Discussion) and the Conclusion.

Residual heavy-tailed patterns persist in extreme years; future research will refine jump-threshold calibration and employ event-based samples for enhanced robustness.

**Comment 2**

Reliance on annual panel data excludes high-frequency dynamics.

Response:

Addressed in the Conclusion.

We note that annual aggregation can smooth intra-year volatility and propose the use of quarterly or event-window data to better capture high-frequency wave–particle transitions.

Comment 3

Practical recommendations for firms and regulators are too broad and lack detailed guidance.

Response:

Expanded in the Conclusion, now offering concrete recommendations:

•	Firms should monitor ROA–ROE divergence as an early-warning signal of structural imbalance;

•	Regulators may track jump proportions as indicators of sectoral stress;

•	Investors can incorporate jump risk alongside volatility in portfolio allocation.

**Comment 4**

The causal mechanisms behind the link between macroeconomic events and jump dynamics are not rigorously tested.

Response:

The Conclusion clarifies that current analyses are correlational and proposes future application of instrumental-variable and event-study designs to identify causal relationships.

**Comment 5**

Future research directions are presented vaguely without clear methodological pathways.

Response:

The Conclusion now outlines four explicit extensions:

(1) adoption of higher-frequency data,

(2) cross-industry validation,

(3) integration of behavioral-finance perspectives, and

(4) AI-driven interpretable modeling (e.g., contrastive GNNs, attention-based LSTMs).

**Comment 6**

Heavy reliance on physics analogies may risk conceptual oversimplification.

Response:

We sincerely appreciate this insightful comment.

In the revised manuscript, we have **clarified that the “wave–particle duality” is not used as a literal physical analogy but as a conceptual framework** that integrates continuous (wave-like) and discrete (particle-like) dynamics in corporate finance.

Specifically, **in Section 2.3 (Interdisciplinary and Complex Systems Approaches) and the last paragraph of Section (Conclusion)**, we emphasize that this framework serves as a modeling metaphor rather than a physical parallel.

Its purpose is to unify two empirically observed states of financial behavior—

(1) gradual, continuous fluctuations (e.g., operational efficiency changes in ROA), and

(2) sudden, discrete jumps (e.g., policy or restructuring shocks affecting ROE).

This clarification reduces the risk of oversimplification and instead highlights the interdisciplinary advantage of using physical metaphors to describe complex adaptive financial systems.

The revised version thus presents the duality framework as an integrative modeling lens—consistent with Reviewer #2’s caution—rather than a literal application of quantum mechanics.
