# Supplementary material for: The wave-particle duality of corporate financial metrics
Source: PLoS One. 2025 Nov 21;20(11):e0336976. doi: 10.1371/journal.pone.0336976 (PMC12637925; doi:10.1371/journal.pone.0336976)
Supplement: Attachment — Submitted filename: Decision Letter to Authors.pdf [file pone.0336976.s004.pdf]

Decision Letter to Authors

Manuscript ID: PONE-D-25-40877R1
Title: *The Wave–Particle Duality of Corporate Financial Metrics*

Dear Authors,

Thank you for submitting the revised version of your manuscript to *PLOS ONE*. I appreciate the time and effort you devoted to addressing the reviewers' and editor's comments with clarity and detail.

After carefully evaluating your revision and responses, I am pleased to accept your manuscript for publication in *PLOS ONE*.

Congratulations to you and your co-authors on this achievement, and thank you for choosing *PLOS ONE* as the venue for your research.

With best regards,

Dr. Dipendra Karki
Academic Editor
PLOS ONE
